# Acute total hip arthroplasty for anterior hip fracture–dislocation: a case report

**DOI:** 10.1093/jscr/rjag138

**Published:** 2026-03-11

**Authors:** Shunsuke Ikezawa, Tomofumi Nishino, Natsuo Yoneda, Kentaro Tayama, Hiroshi Noguchi

**Affiliations:** Department of Orthopaedic Surgery, Japanese Red Cross Mito Hospital, Mito, Japan; Department of Orthopaedic Surgery, University of Tsukuba Hospital, Tsukuba, Japan; Department of Orthopaedic Surgery, Institute of Medicine, University of Tsukuba, Tsukuba, Japan; Department of Orthopaedic Surgery, Japanese Red Cross Mito Hospital, Mito, Japan; Department of Orthopaedic Surgery, Japanese Red Cross Mito Hospital, Mito, Japan; Department of Orthopaedic Surgery, Japanese Red Cross Mito Hospital, Mito, Japan

**Keywords:** anterior hip fracture–dislocation, acute total hip arthroplasty, fix and replace

## Abstract

Hip fracture–dislocations accompanied by femoral head or acetabular fractures remain challenging, and the optimal treatment strategy is still controversial. Anterior hip fracture–dislocations are particularly rare, and reports describing acute total hip arthroplasty (THA) for this injury pattern are limited. We report the case of a 74-year-old active woman who sustained an anterior hip fracture–dislocation with femoral head and acetabular anterior wall fractures after a low-energy fall. The injury was classified as Pipkin type IV and Brumback type 4B. Following closed reduction, acute THA was performed via a posterior approach using a dual mobility cup without additional internal fixation, as adequate acetabular coverage and primary stability were achieved intraoperatively. Immediate full weight-bearing was allowed. At 6 months postoperatively, the patient had resumed recreational sports, with a Harris Hip Score of 92 and no complications. Acute THA may be a viable treatment option for selected anterior hip fracture–dislocations.

## Background

Hip fracture–dislocations accompanied by femoral head and/or acetabular fractures remain challenging injuries, and an optimal treatment strategy has not yet been clearly established. Even when open reduction and internal fixation (ORIF) is performed to preserve the femoral head, secondary complications such as post-traumatic osteoarthritis (~20%), heterotopic ossification (16.8%), and avascular necrosis of the femoral head (11.9%) are frequently reported, often necessitating conversion to total hip arthroplasty (THA) [1].

Anterior hip dislocations account for ~10% of all hip dislocations and are far less common than posterior dislocations. Cases complicated by associated fractures requiring surgical intervention are particularly rare, and clinical reports remain limited. We herein report a rare case of anterior hip fracture–dislocation with concomitant femoral head and acetabular fractures treated successfully with acute total hip arthroplasty (acute THA).

## Case presentation

A 74-year-old woman presented after slipping during a bowling approach and falling onto the lateral aspect of her right thigh with the hip in a flexed, adducted, and externally rotated position. She experienced severe hip pain and inability to move. Her medical history included hypertension, dyslipidaemia, Graves’ disease (status post-thyroidectomy), and vertigo. She was highly active, regularly participating in table tennis, tennis, and bowling.

On admission, she was alert and haemodynamically stable. Physical examination revealed mild external rotation and leg-length discrepancy of the right lower limb, with tenderness in the femoral triangle. Distal neurovascular status was intact. Laboratory findings were unremarkable, and bone mineral density was within the normal range.

**Figure 1:**
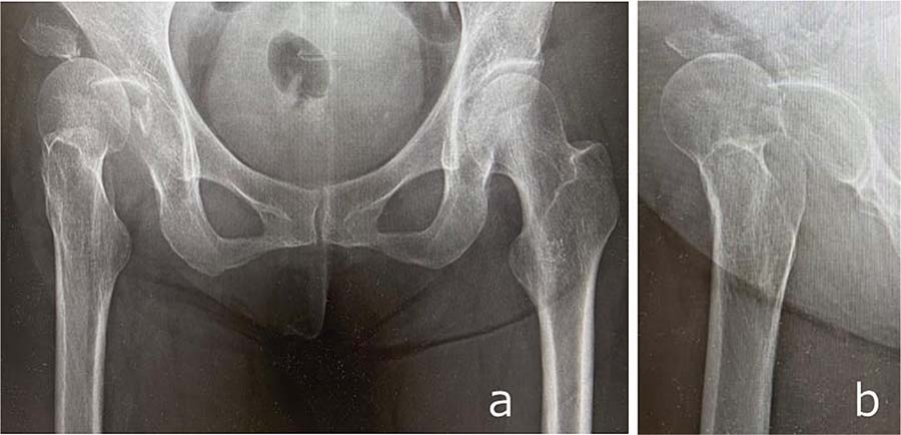
Anteroposterior (a) and lateral (b) radiographs of the right hip obtained at initial presentation, demonstrating anterior hip dislocation associated with acetabular and anterior inferior iliac spine fractures.

**Figure 2:**
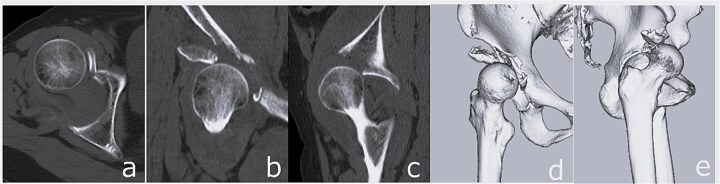
CT images obtained at initial presentation: Axial (a), coronal (b), and sagittal (c) views, as well as three-dimensional reconstructions (d and e), showing anterior hip dislocation with fractures of the acetabular anterior wall and anterior inferior iliac spine.

Plain radiographs demonstrated an anterior dislocation of the right hip with associated acetabular fractures (Fig. 1). Computed tomography (CT) after closed reduction revealed fractures of the anterior inferior iliac spine (AIIS), the anterior acetabular wall, and an intra-articular femoral head fracture fragment (Fig. 2). The injury was classified as Pipkin type IV [2] and Brumback type 4B [3].

**Figure 3:**
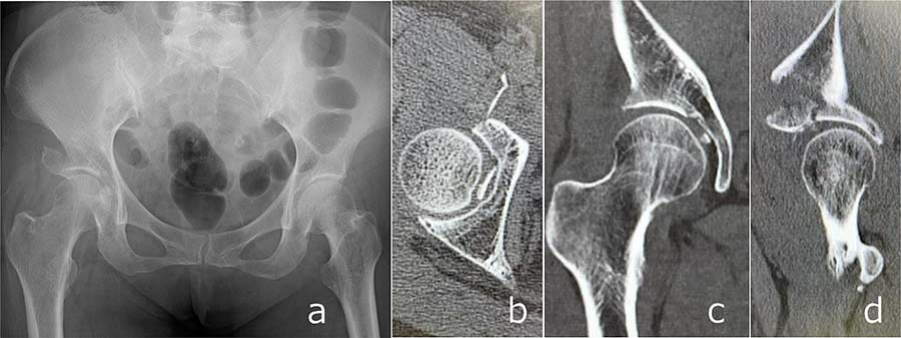
Post-reduction imaging: Anteroposterior radiograph (a) and CT images (b–d) demonstrating reduction of the hip joint with an intra-articular femoral head fracture fragment and anterior acetabular wall involvement.

Closed reduction was performed under sedation. Post-reduction imaging showed incarceration of the femoral head fragment within the acetabulum and partial deficiency of the anterior roof arc (Fig. 3). Acute THA was scheduled 7 days after injury. Preoperative planning was based on careful evaluation of three-dimensional CT images. Although a dedicated three-dimensional templating system was not available at our institution, fracture lines, bone defects, and the expected acetabular coverage were assessed visually using multiplanar and three-dimensional reconstructed CT images. Based on this assessment, we anticipated that sufficient rim engagement and primary stability of a cementless cup could be achieved without additional internal fixation.

**Figure 4:**
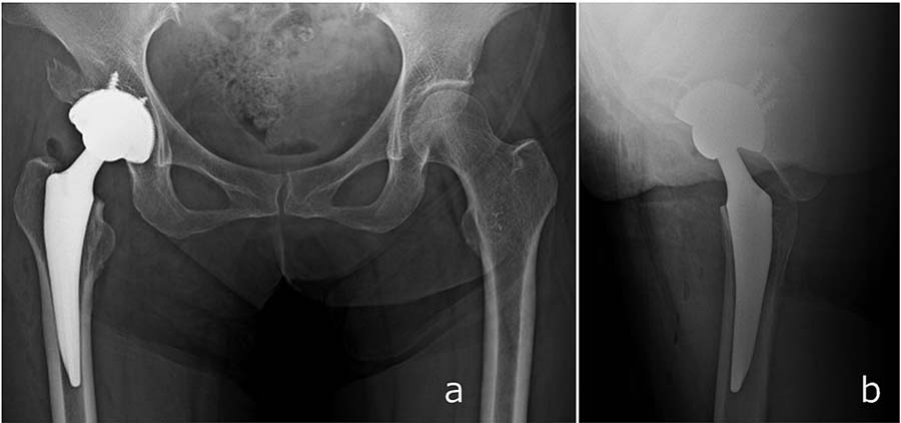
Immediate postoperative anteroposterior (a) and lateral (b) radiographs demonstrating stable placement of the total hip arthroplasty components.

Surgery was performed via a posterior approach. The femoral head fragment avulsed at the ligamentum teres was found incarcerated within the acetabulum. Although fractures of the AIIS and anterior wall were identified, sufficient acetabular coverage and stable press-fit fixation of a cementless cup were achieved without additional internal fixation. A dual mobility cup (DMC) and a cementless femoral stem were implanted (Fig. 4).

**Figure 5:**
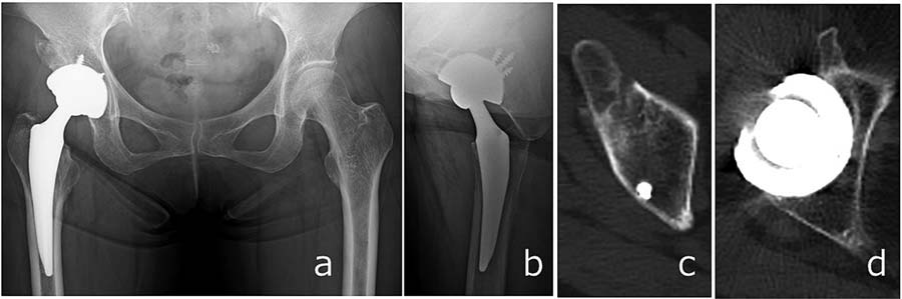
Radiographs (a and b) and CT images (c and d) obtained at 6 months postoperatively, showing stable implant fixation and partial union of the anterior inferior iliac spine and acetabular anterior wall fractures.

Full weight-bearing was allowed from postoperative day 1. The patient regained independent ambulation and was discharged home on postoperative day 28. At 6 months, she had resumed recreational activities with a Harris Hip Score of 92. No complications were observed, and follow-up CT demonstrated healing of the anterior wall fracture and partial union of the AIIS fragment (Fig. 5).

## Discussion

ORIF has traditionally been regarded as the standard treatment for hip fracture–dislocations to preserve the native femoral head. However, in cases with severe displacement, comminution, or associated acetabular fractures, achieving anatomical reduction is technically demanding, and the risk of late complications remains substantial [1]. As a result, the concept of ‘fix and replace,’ incorporating THA during the initial surgery in selected patients, has gained increasing attention [4].

Clinical outcomes of femoral head fractures vary according to the Pipkin classification. While relatively favourable results have been reported for Pipkin types I and II, outcomes for type IV injuries are consistently poor [2]. Engel *et al*. reported high rates of post-traumatic osteoarthritis and conversion to THA following ORIF for Pipkin type IV fractures [5]. Menger *et al*. identified Pipkin type III/IV fractures, acetabular comminution, articular impaction, advanced age, and osteoporosis as predictors of poor outcomes after ORIF, recommending consideration of acute THA in such cases [6]. Yoon *et al*. also demonstrated that acute arthroplasty reduced the need for secondary THA in severe femoral head fractures [7].

The present case is notable because it involved an anterior fracture–dislocation, a pattern that is rare and poorly represented in the literature compared with posterior dislocations [8]. Anterior dislocations are often associated with disruption of anterior stabilizing structures, which may increase the risk of postoperative instability.

In this patient, the combination of a Pipkin type IV fracture, Brumback type 4B injury, thin articular fragments unsuitable for stable fixation, advanced age, and high functional demands supported the decision to perform acute THA rather than ORIF.

Intraoperative judgment was considered crucial in determining the need for additional fixation. A three-dimensionally porous-coated cementless cup was selected to maximize initial fixation. In anticipation of insufficient primary stability, a reinforcement ring was prepared as a backup option. Ultimately, stable press-fit fixation was achieved without supplemental fixation, and therefore additional implants were not required. A key consideration was whether additional internal fixation of the anterior wall and AIIS fractures was necessary. In hip fracture–dislocations accompanied by acetabular fractures, the indication for fixation during acute THA remains controversial and should be guided by the ability to achieve stable acetabular component fixation rather than fracture morphology alone. Recent studies suggest that internal fixation is not mandatory when sufficient acetabular coverage and primary cup stability can be achieved intraoperatively [9, 10]. In the present case, adequate rim engagement and press-fit stability were confirmed, and the fracture fragments did not compromise the weight-bearing dome.

Additional fixation would have required extensive anterior exposure, increasing surgical invasiveness and the risk of complications in elderly patients. Given the thin, non-reconstructable nature of the fracture fragments, omission of internal fixation was considered reasonable, supported by subsequent radiographic healing and favourable clinical outcome.

Another important aspect was implant selection. Anterior fracture–dislocations involve disruption of anterior capsuloligamentous structures, potentially predisposing patients to postoperative instability. DMCs have been shown to reduce dislocation risk in high-risk populations and in trauma-related THA [11]. In this context, the increased jump distance and enhanced stability provided by a dual mobility construct were advantageous.

The posterior surgical approach was deliberately chosen to avoid further disruption of the already injured anterior soft tissues. The combination of a posterior approach and a DMC allowed preservation of remaining stabilizing structures while maximizing implant stability. The absence of postoperative dislocation and excellent functional recovery support this strategy.

Although evidence specific to anterior hip fracture–dislocations treated with acute THA remains limited, this case suggests that acute THA without additional internal fixation can be a safe and effective option when careful intraoperative assessment confirms sufficient acetabular stability.

## Patient perspective

The patient was satisfied with the treatment outcome and recovery process.

## Approval of the research protocol

This study was conducted in accordance with the declaration of Helsinki. This study was conducted with approval from the Ethics Committee of Japanese Red Cross Mito Hospital.
